# Modified Fontan conversion: an alternative technique for grown-up patients

**DOI:** 10.1186/1749-8090-10-S1-A252

**Published:** 2015-12-16

**Authors:** María-Teresa González-López, Ana-María Pita-Fernández, Juan-Miguel GilJaurena, Ramón Pérez-Caballero-Martínez, Raquel Yotti-álvarez, Raquel Prieto-Arévalo, Fernando SarnagoCebada

**Affiliations:** 1 Paediatric Cardiac Surgery Department, Gregorio Marañón Hospital, Madrid, Spain; 2 Grown-up Congenital Patients Unit, Cardiology Department, Gregorio Marañón Hospital, Madrid, Spain

## Background/Introduction

A need persists for Fontan conversion (classical Fontan to total cavopulmonary connection (TCPC)) that provides alternative approaches for the individual anatomical challenges occurred in these unusual and complex congenital grown-up patients.

## Aims/Objectives

We describe a novel bail-out technique for Fontan conversion.

## Method

A 28-year-old woman (tricuspid atresia/Blalock-Taussing shunt) underwent atriopulmonary Fontan procedure at the age of 3. At 25-year follow-up, she developed a progressive worsening in functional class and palpitations, despite antiarrhythmic treatment. Imaging studies showed no stenosis in the atriopulmonary connection. She underwent Fontan conversion. The right atrium (RA) was extremely dilated and the right pulmonary artery (RPA) was noted to have a striking small diameter/short length along with a dilated IVC. The RA was downsized, the atriopulmonary Fontan was disconnected and cryo-Cox-Maze IV procedure was carried-out. After cooling the patient to 28°C, ventricular fibrillation was induced (12 minutes) and carbon dioxide field flooding was employed to create an atrial septal defect. The RA was closed and the TCPC was performed (24-mm Goretex conduit): the proximal end-to-end anastomosis to the inferior-venae-cavae was done. The distal anastomosis (conduit-to-RPA) was desestimated due to the size mismatch. An end-to-end anastomosis between the main PA and the conduit was then done followed by the end-to-side anastomosis of the superior-venae-cavae (SVC) to the conduit. The patient was weaned from cardiopulmonary bypass (307 minutes) uneventfully. Epicardial leads and dual-chamber pacemaker were implanted.

## Results

At 10-month follow-up, she remains in synus rhythm/asymptomatic. A computed tomography angiography has showed an optimal flow into the Fontan circuit.

## Discussion/Conclusion

The Fontan conversion is an unusual procedure in which the standard distal anastomosis is performed to the RPA, but our surgical design includes a two-anastomosis modification (extracardiac conduit-to-main PA and SVC), allowing the matching of the curved conduit to the PA when the RPA is not adequate. More patients may be candidates for this surgery and modifications of the standard technique are mandatory.

## Consent

Written informed consent was obtained from the patient for publication of this abstract and any accompanying images. A copy of the written consent is available for review by the Editor of this journal.

